# Critical nursing and health care aide behaviors in care of the nursing home resident dying with dementia

**DOI:** 10.1186/s12912-019-0384-5

**Published:** 2019-11-29

**Authors:** Genevieve N. Thompson, Susan E. McClement

**Affiliations:** College of Nursing, Rady Faculty of Health Sciences, University of Manitoba, Winnipeg, MB R3T 2J5 Canada

**Keywords:** Expert nursing, Nursing home, Dementia, Palliative care, End of life care, Health care aide

## Abstract

**Background:**

With the aging of the population, dying with dementia will become one of the most common ways in which older adults will end their final years of life, particularly for those living in a nursing home. Though individuals living with dementia have complex care needs and would benefit from a palliative approach to care, they have traditionally not been recipients of such care. An important aspect of determining quality in end-of-life care is the identification of expert practices, processes or behaviors that may help achieve this care. However, for those living with dementia in nursing homes, we have a limited understanding of how to best support expert end of life care. To redress this gap in knowledge, the purpose of this study was to examine and describe expert care of the individual with dementia approaching death from the perspective of nurses and health care aides (HCAs) identified by their peers as having special expertise in caring for this population.

**Methods:**

A qualitative research design known as Interpretative Description was used to conduct the study. Expert nurses and HCAs were identified through a two-phase nomination process. Individual semi-structured interviews were conducted with consenting participants. Data were analyzed using constant comparative analysis to determine the key critical behaviors.

**Results:**

Analysis of data collected from expert nurses (*n* = 8) and HCAs (*n* = 7) revealed six critical behaviors when caring for residents dying with dementia. All nurses and HCAs unanimously endorsed that the overarching goal of care is similar for all residents who are actively dying; to achieve comfort. The six critical behaviors in caring for residents dying with dementia included: 1) recognizing and responding to changes in a resident’s pattern of behavior; 2) attending to the person; 3) working with the family; 4) engaging with others; 5) responding after the death has occurred; and 6) having a positive attitude toward care of the dying.

**Conclusions:**

The critical behaviors described by nurses and HCAs in this study provides emerging evidence of best practices in care of those with dementia and their families, particularly near the end of life within the nursing home setting.

## Introduction

With the aging of the population, dying with dementia will become one of the most common ways in which older adults will end their final years of life [1]. Though individuals living with dementia have complex care needs and would benefit from a palliative approach to care, they have traditionally not been recipients of such care. There is increasing recognition that a palliative approach to care is a vital and integral part of all clinical practice, regardless of illness, its stage of progression, or the context in which the care is provided [2]. Individuals dying with dementia have significant threats to their physical, social, psychological, and spiritual health [3–5]; areas amenable to a palliative approach to care. Owing to the cognitive deficits and functional impairments that occur as dementia progresses, many individuals living with dementia will require formal care from facilities such as nursing homes. Nursing homes (NHs) are residential facilities that provide care for those with chronic illness, disability, mobility problems, and cognitive decline [6]. In Canada, 87% of NH residents have a diagnosis of dementia [7] and research indicates these individuals require 36% more nursing care than those without the disease [8]. Clearly, as increasing numbers of people with dementia live out their final days in NH settings, staff in these facilities will be required to provide quality end-of-life care for this resident population.

An important aspect of determining quality in end-of-life care is the identification of expert practices, processes or behaviors that may help achieve this care. While the delivery of health care is optimally provided by a multidisciplinary team, it is nurses and health care aides (HCAs) who are omnipresent in the NH setting. An estimated 80 to 95% of direct resident care is provided by HCAs and nurses play central roles in making care decisions for NH residents, including those at end of life [9]. Research exploring expert nursing behaviors in the care of the dying has been conducted in adult intensive care settings [10] and more broadly across nursing practice [11]. Research generally in NHs is limited and has not focused specifically on the unique skills needed in expert care of the resident dying with dementia [12]. Our understanding of care behaviors for this population is therefore incomplete. To redress this gap in knowledge, the purpose of this study was to examine and describe expert care of the individual with dementia approaching death from the perspective of nurses and HCAs identified by their peers as having special expertise in caring for this population. Their close proximity and involvement in resident care provides them with a unique perspective that can foster our understanding of quality end-of-life care in the NH, and of the behaviors that help achieve it.

## Methods

A qualitative research design known as Interpretative Description [13] was used to conduct the study between January 2014 and March 2015. Interpretive description is an approach that enables the researcher to investigate clinical phenomena of interest about which little is known, with the objective of developing a coherent conceptual description useful for clinical practice [14]. Given the paucity of research exploring the expert care at the end of life for persons living with dementia provided by nurses and HCAs working in the NH setting, the use of interpretive description is both justified and warranted. This study was approved by the Education/Nursing Research Ethics Board at the University of Manitoba.

### Recruitment

Participants were recruited from across five NHs in a central Canadian province using a purposive sampling technique. These NHs volunteered to participate in the study after a presentation by the first author at the monthly meeting of the regions’ 39 directors of care and represent a mix of proprietary (*n* = 2), medium bed size (*n* = 3; bed size 150), and facilities with specialty dementia care units (*n* = 3).

Expert nurses and HCAs were identified through a two-phase nomination process developed and used successfully in studies of expert nursing practice [10, 12, 15]. In the first phase, the lead author and study nurse held a staff meeting to introduce and describe the study at each NH facility from which participants were drawn. Attendees at the meeting received a study information sheet and a nomination form to guide them in the identification of nurses and HCAs deemed experts in the care of older adults dying with dementia. The nomination form consisted of three descriptors of health care provider experts, along with the particular skills identified from the literature as being important when providing care to dying residents with dementia. Descriptors asked respondents to identify individuals who: 1) had the clinical expertise that enabled them to determine the need to transition a resident’s care from curative/restorative to palliative care; 2) were skilled at identifying and responding to the needs of dying residents and their families; and 3) demonstrated expertise in responding to the cognitive and behavioral changes of residents dying with dementia, and the ability to communicate effectively with their families.

Nurses and HCAs could participate by anonymously writing the names of up to three colleagues who best fit the descriptors provided and depositing their completed nominations in a locked ballot box located in a central location within each of the five participating NH facilities. Individuals could nominate themselves if they felt they met the descriptors. Extra information sheets and nomination forms were distributed to all nursing units for staff who were unable to attend the meeting. After approximately 2 weeks, the research nurse returned to the facility to collect the ballot box containing the completed nomination forms.

In phase 2, nominations were counted and individuals with three or more nominations for each descriptor were sent a letter of invitation through their place of employment to participate in a face-to-face semi-structured interview with the research nurse. A follow-up letter was sent to those individuals who did not reply to the original letter within 2 weeks.

### Semi-structured interview

After providing written informed consent, nominated expert HCAs and nurses completed a demographic form that captured their gender, age, years of clinical experience, years of working in the current facility, and any additional education they had taken in dementia or palliative care. They then participated in individual semi-structured interviews where they were invited to describe the behaviors they deemed critical in caring for dying residents with dementia. Participants were asked to recount the most recent incident they could remember in which they provided care to this type of resident to reflect upon any perceived differences in caring for residents dying with dementia compared to those who were not; and to describe the salient features of a “good death” for a resident with dementia. Participants were also asked to share examples in which colleagues had displayed both positive and negative attitudes in caring for patients dying with dementia and their families. Interviews lasted approximately 60 min and were conducted in a mutually convenient location. Each interview was digitally-recorded and transcribed verbatim by a transcriptionist.

### Data analysis

Demographic data collected from participants was analyzed using descriptive statistics. Transcribed interviews were checked against the recorded interview by the research nurse to ensure accuracy. Constant comparative content analysis, the process of identifying, coding, and categorizing patterns in the data, was used to analyze the data [16]. Transcripts were first read in their entirety in order to gain a sense of the whole interview and to identify key words, phrases, or emerging themes that may form codes. Next, categories of behaviors were developed by clustering coded data into meaningful groups. The basic properties of these categories were then defined, and relationships between categories identified. Finally, categories were compared to ensure they were mutually exclusive [17]. Descriptions of each category and their relationships with each other were written to provide a coherent picture of expert care of the dying. Analysis of nurse and HCA data was done separately and then examined for similarities and differences in behaviors. Data coding was conducted by both authors who engaged in discussion to reach consensus on the determination of code labels.

Our goal in conducting this study was present a truthful representation of the voice and experience of nurses and health care aides with recognized expertise in caring for residents with dementia who are dying. Adherence to procedures described by Lincoln and Guba [18] as regards principles of credibility, dependability, confirmability, were used to facilitate the rigor of our work. Credibility was addressed by taking time to establish a rapport with study participants before interviews began, demonstrating empathy during interviews when challenging experiences were being shared, and checking with participants during the interview to confirm understanding of what was being said.

Detailed minutes of all data analysis meetings were kept to capture data collection and analysis procedures, a description of participant demographics, and the use of direct participant quotations to represent the findings were approaches used to ensure the criteria of confirmability and dependability. Rich description of the phenomnenon of interest provided by participants and clear, detailed description of study processes and procedures such that replication of the work could be conducted address the criterion of transferability.

## Results

The nomination process identified 23 nurse and 20 HCA experts from across the five NH facilities. Of these, 8 nurses and 7 HCAs agreed to be interviewed. Nominees declined participation due to a lack of either time or interest. As described in Table 1, nurses comprised both registered nurses and licensed practical nurses with nearly 20 years of health care experience on average. HCAs reported similar length of experience.

**Table 1:** Participant Demographics

Variable	n (%)	
Gender		
Female	15 (100)	
Male	0	
Experience in Years	Nurses (mean)	Health Care Aides (mean)
Personal Care Home	14.88	13.84
Geriatrics	19.13	14.15
Total Health Care Experience	19.69	13.84
Professional Designation		
Registered Nurse	4(27)	
Licensed Practical Nurse	4(27)	
Health Care Aide	7(47)	
Education Completed		
Baccalaureate Degree in Nursing	1 (7)	
Registered Nurse Diploma	3 (20)	
Licensed Practical Nursing Diploma	4 (27)	
Health Care Aide Certification	7(47)	
Employment Status		
Full-time	11(73)	
Part-time	4(27)	
Specialized Palliative Care Course		
Yes	7(47)	
No	8(53)	
Dementia Care Training (*n* = 14)		
Yes	12(86)	
No	2(14)	
Hospice and Palliative Care Nursing Certification (Nurses only; *n* = 8)		
Yes	0	
No	8(100)	
Gerontological Nursing Certification (Nurses only; *n* = 8)		
Yes	2(25)	
No	6(75)	

Analysis of data collected from expert nurses and HCAs revealed six critical behaviors when caring for NH residents dying with dementia (See Table 2). In many instances, nurses and HCAs described that the care they provided to residents did not change significantly because of a diagnosis of dementia:

*“It’s just they’re human, you know. They need the same love and care as everybody else does. And just because somebody has something [like dementia], like I’d never treated them differently.”* [HCA04: 401–403]

**Table 2:** Critical behaviors of nurses and healthcare aides in caring for nursing home residents dying with dementia

*Behaviors*	Characteristics	*Operational definitions*	
		*Positive*	*Negative*
Recognizing and responding to changes is resident’s pattern of behavior	a) Use of knowledge and skills in dementia and palliative care; b) Integration of knowing resident’s normal way of being with changes being witnessed; c) Altering plan of care based on assessment	Behaviors that recognize and act on changes in resident’s normal patterns of behavior Behaviors that recognize dementia is a terminal illness	Behaviors that fail to acknowledge the significance of the changes in resident’s pattern of behavior Behaviors that continue with same approach to care
Attending to the person	a) Acknowledge individuality; b) Therapeutic use of self; c) Provision of physical and psychological comfort	Behaviors that honor the individuality of the resident Behaviors that convey an emotional connection toward the resident Behaviors that seek to achieve and maintain physical and psychological comfort	Behaviors that lack of honoring of the individuality of the resident. Avoidance of dying residents Poor symptom management because of a poor knowledge base
Working with family	a) Normalizing dementia and dying; b) Decreasing potential for future regret; c) Keeping family comfortable while sitting vigil	Behaviors that reduce potential for future regret Behaviors that respond to family’s need for information Behaviors that maintain family involvement and respect expertise of the family Behaviors that assist with family member’s comfort	Behaviors that exclude family as part of unit of care Behaviors that are reactive rather than proactive Passing judgement on family decisions and family behaviors toward the resident
Engaging with others	a) Getting help; b) Teamwork	Behaviors that actively engage those best able to meet the needs of the resident	Behaviors that exclude others from the care of the resident Behaviors that maintain hierarchy
Responding after the death has occurred	a) Spending time with resident and family; b) Remembering and celebrating resident through storytelling c) Rituals (I think this needs a bit more specificity. Rituals that are family/culturally sensitive and specific d) What about supporting other staff here?	Behaviors that demonstrate respect toward the resident and family Behaviors that honor and remember the resident Behaviors that provide emotional support for self, colleagues, and family members	Avoidance behaviors
Having a positive attitude towards care of the dying	a) Advocate; b) Mentor; c) Bearing witness	Behaviors that demonstrate the health care provider has defined personal role in care for dying	Behaviors that show a lack of confidence in care for dying

Earlier in the disease course, experts explained that they may have had to take a different approach to care to overcome some of the challenges presented by the presence of dementia. For instance, nurses and HCAs may need to respond to changes in the resident’s verbal communication abilities or navigate aggressive behaviors exhibited by a resident resulting from a shifting understanding of the intentions of those who provide their care. However, all nurses and HCAs unanimously endorsed that the overarching goal of care is similar for all residents who are actively dying; to achieve comfort:

*“And to make sure that their passing is done with respect and dignity. To help them realize that they’re not alone. Somebody is there with them. And you know what, it’s not uncommon for staff to take shifts sitting with residents when they’re making their transition… And especially because they are part of your family after a while…You get very familiar and close to people. You get used to their ways. And even with people who have dementia, you get used to their ways, it’s sometimes very, very sad when they pass on. Sometimes for the staff it feels like a blessing because you knew the person was suffering so it’s a blessing. But still a sad moment. I think it’s important, and I really believe that staff do it to keep that person to give them comfort, not only physically but emotionally and spiritually.*” [RN012: 542–556]

The six behaviors emerging from the data are detailed below, supported by exemplars.

### Recognizing and responding to changes in a Resident’s pattern of behavior

Nurses and HCA experts were able to identify changes in a resident’s normal pattern of behavior and recognize when such changes signaled a transition to end of life. All study participants endorsed the critical importance of assessment in the detection of significant changes in the resident, characterizing this activity as ‘keeping an eye on’ [HCA04: 569] and regularly ‘checking up on’ [HCA03: 1355] residents. HCA knowledge of residents’ normal behavior was gleaned as a result of caring for residents over long periods of time. Expert HCAs in this study were able to cue into and discern the difference between normal day-to-day variations in such things as the resident’s ability to swallow or ambulate, and tendencies toward aggressive behavior, versus those changes that heralded the start of the resident’s decline toward death:

*“Well, I think that they, it probably starts with their mobility because a lot of people I find that have dementia are usually walkers. Usually. And it usually is their mobility that deteriorates first. And then they’ll be in a wheelchair and then they’ll, then after, you know, progression of time, they usually just maybe a week or two or maybe a month or so, will be in bed, you know. And then they don’t want to eat. And then they don’t want to get up. And it’s just, you know, day after week after of that. And, yea, I just, I just find that they may not be able to tell you anything of what’s wrong. But, you get used to the person so you can just sort of tell.”* [HCA011: 502–511]

Communicating these changes to the nurse was an important follow-up step in the process. Nurses observing or receiving reports about significant changes in a resident’s status worked to actively integrate pertinent findings together to understand what was happening. They accomplished this by drawing on their knowledge of both dementia care and palliative care practices, and their familiarity with what to expect as death draws near. Often this required piecing together signs of subtle changes in the resident’s normal behavior that heralded decline. This work of ‘piecing together’ required that nurses and HCAs use knowledge gleaned from experience about the individualized and varied ways in which symptoms can present that was “beyond the textbook signs” [LPN06: 860]. As another nurse described:

*“And I think that because when people are often advanced in their dementia processes as a lot of our residents are, nurses and health care aides aren’t always fantastic about picking up on the cues for pain….So trying to deal with some pain and symptom management issues when their symptoms aren’t textbook and aren’t easily recognizable by everyone. I think that’s my greatest challenge.”* [RN02: 21–36]

Both HCA and nurse experts stressed the necessity of moving beyond reliance on verbal reporting of discomfort by residents with dementia, and watching instead for non-verbal signs. HCAs looked to the behavioral cues of residents to direct their care, enabling them to “know they are in pain by a look” [HCA01: 199]. Nurses also identified that they “listened to non-verbal cues” [RN012: 208] and that the assessment of discomfort was done through attending to visual cues:

*“I find that those that have a mild form of dementia are usually, more vocal in what’s happening. They can tell you regarding any pain issues that they are having. You’ll notice deterioration in… communicating with them. You’ll notice a lot of verbal cues versus the ones that have advanced dementia you’ll notice their body failing more. So their body will give you more cues than they will verbalize.”* [RN05: 6–13]

By recognizing the salience of their assessment findings, experts were able to be proactive in setting a course aimed at achieving a good death for the resident.

### Attending to the person

Nurses and HCAs exemplified three core behaviors that supported this theme. These behaviors required that they: (1) acknowledged the individuality of each resident; (2) engaged in therapeutic use of self; and (3) provided physical and psychological comfort.

Central to acknowledging the individuality of each resident was that nurses and HCAs viewed the resident as a whole person. To achieve this, they maintained an awareness that the person the resident was prior to their diagnosis of dementia was still present in some way. One approach to caring for the whole person included respecting the likes and dislikes of the resident. Nurses and HCAs then integrated knowledge of resident preferences and personalized their care based on it. Care became less task focused and therefore, required a level of flexibility and creativity:

*“She loved Pepsi. So her, on her deathbed we wet the toothette with Pepsi, you know, just so, here you go, your last kick at the can kind of thing, right. And her family cried that we even thought to do something like that.”* [RN05: 886–888]

Caring for the resident as a whole person also involved both nurses and HCAs continuing to talk and relate to the resident, even when the resident could no longer verbally respond to them. This behavior was important prior to and during the provision of even basic care activities:

*“Still saying step by step this is what I’m going to do and this is why I’m doing it even if you’re not knowing how much they’re really comprehending of what you’re saying.”* [RN014: 153–155]

In addition to acknowledging the individuality of the resident, nurses and HCAs engaged in therapeutic use of self when providing care. Experts identified the importance of being ‘present’ when interacting with residents and providing care:

*“And just to offer yourself to them because that’s often all you can do. So sometimes people die with no medical issues. And as nurses we’re often very medically focused, right. So it’s just switching gears and allowing yourself to just be able to be OK with doing nothing, and knowing that doing nothing is doing so much.”* [RN02: 1292–1303]

Therapeutic use of self also meant that experts engaged in active listening and made emotional connections with residents. Nurses and HCAs were not afraid to show caring through the use of touch; holding hands, giving hugs, a touch on the shoulder. In using the self, a caring and safe space was created, setting a tone of care that put the resident first and foremost:

*“Being there is being present and giving your whole active attention to being present. And often that means touching. If it’s a person who has never really like to, say hold hands or hugging or things like that, I wouldn’t disrespect them by doing that at that point in time. But I might stroke their arm and just say, I’m here with you, you know, and just, if it’s a person who’s enjoyed books all their lives, you know, to sit and read a little bit.”* [RN012: 59–65]

In the last component of attending to the person, experts engaged in behaviors designed to provide residents with physical and psychological comfort. For HCAs, attending to the body of the resident is the thrust of their care. Experts spoke about the important care activities in keeping the resident “clean” such as bathing, turning, repositioning, performing mouth care, keeping them dry and cool, ensuring they are in comfortable clothes, combing hair, and even putting on make-up. For example, *“every two hours we check. We do this. The resident we comb her hair, we make her make-up. We make her face. Mouth care.”* [HCA10: 1075–1076]. However, this physical care was not divorced from psychological comfort or use of self. HCAs that demonstrated expert behaviors seamlessly wove task-type care with attention to, and respect for, the individual. In the end of life, expert HCAs saw their presence as being comforting to the resident.

HCAs recognized the fragility of the resident as they drew closer to death and placed importance on always ensuring care was gentle and not rushed. This was particularly important when HCAs were working together in providing care, for instance, “working as a pair team when turning”. [HCA04: 175]. For nurses, physical comfort was an important goal, particularly when symptoms such as pain or dyspnea were present. Nurses described the importance of aggressive pain management to achieve physical comfort:

*“I don’t stop. I refuse to have anyone die in pain. Whether they’re under my care or not. Whether I’m responsible for them or not, I refuse, as a nurse, to have anyone die in pain. Because, in this day and age, we have the technology that could have someone transition into death at a peaceful, beautiful kind of experience and, whether I have to pull teeth or sit on someone, I know at the end of the day, I can go home to my family and know I’ve done a good thing.”* [RN05: 579–585]

Experts promoted psychological well-being by such actions as speaking to residents in calming tones, playing music, and being a familiar presence. In many instances, nurses and HCAs expressed how caring for someone dying with dementia was easier than caring for someone without dementia because they did not have to as engage in discussion with the resident about their fears and anxiety surrounding death. Similarly, the spiritual needs of residents with dementia was something that few, if any, participants mentioned as being important to attend to:

*“I suppose the knowledge that maybe they don’t understand what’s going on…they’re past that stage so there’s not as many questions from the patient themselves as to what’s happening. It’s more, if there is family around, then, you can explain that this is, this is where we’re at, this is what you can expect to see.* [LPN013: 154–159]

*“When someone does not have dementia they know they’re palliative and they may be dealing with issues of remorse, regret, and you’re dealing with those kinds of emotional things that they are trying to you know that everybody’s got to get their life in order you know, before they pass away. So you’re dealing sometimes more with that. In somebody with dementia it’s not something that usually gets talked about”* [RN014: 165–172]

### Working with the family

The resident’s family was identified by experts as part of their unit of care and an important source of information regarding the resident. Experts spoke of the value of forging relationships with family to optimize the care of the resident. Nurses and HCAs also recognized that families had their own needs and they too required supportive care when the resident was dying. Three categories emerged in relation to working with the family and included behaviors that: [1] normalized dementia and dying [2]; reduced the potential for future regret; and [3] kept the family physically comfortable while sitting vigil.

*Normalizing dementia and dying:* The first category was emphasized by both nurses and HCAs who described behaviors that normalized dementia and dying in an attempt to prepare the family for the death of the resident. This was especially important in the face of challenging behaviors:

*“We get close relationships with them too so. And especially when someone has dementia. The family requires so much support throughout. Like if they have inappropriate behaviors, it’s acknowledging those behaviors and reminding the families that there’s nothing that they can do about it and medication might work but it might not. So it’s just letting the family accept those behaviors and recognize that we accept them and they’re not a bad thing. It’s just, it’s life, what it is now for that person.”* [RN02: 398–404]

Finding out what the family knows or understands about dementia and dying in particular, was a technique often described by nurse experts as a means to gauge where the family may be in terms of their understanding. Recognizing that for many families, death is a new and unfamiliar experience, nurses expressed a strong desire to understand “where families are coming from” [RN09: 555]. Nurses listened to families and respected the expertise of the family regarding the resident. A key aspect of this was to engage in active listening as described below:

*“…some families want to know absolutely everything. And some families don’t want to know anything. So I just kind of see where they’re at in their want and need. And I kind of go from there…Listen. Listen. Stop talking and listen. Listen to where they’re at... It’s to be a really empathetic listener. Because sometimes family don’t want you to talk. They don’t want you to explain it. They just want to know that there’s somebody there that’s going to care for mom or dad or auntie or uncle or grandma. That’s it. That’s all they want to know you know. So I think listening is the biggest part. I think it’s the one thing we have the hardest time doing as human beings.”* [LPN08: 1170–1186]

Providing information in small doses and tailoring the content to family needs was also a way to ensure families received the appropriate kind of information regarding the care needs of the resident:

*“OK, I have to kind of be touchy feely with this family. Or I have to get the horns out with this family, you know. Or I have to explain to this son, this daughter or this grandson, you know, three different ways …So it’s very difficult. And I find that each cause is very unique.”* [RN05: 1118–1122]

Several nurses also acknowledged that for some families, hearing the information from someone else on the care team other than them was important to normalize the dying process.

*“It’s lots of discussion with the family. So, you know, sometimes you don’t change the plan of care right away. It’s letting the family absorb what you’re trying to tell them. Often they, depending who they have in their family, if there’s doctors, they don’t want to hear it from us. They want to hear it from the doctor or heard that the doctor agrees with this. Speech language therapy has been amazing with us when people can’t swallow anymore and it’s not safe to feed them, providing that information to the family. So it’s bringing people on, when the initial discussion with the family isn’t well received. So it’s a fine line of finding out who has the best relationship with that family.”* [RN02: 540–556]

Nurses also used ‘show and tell’ or meaningful analogies to speak frankly and directly to families as a means of ensuring their expectations matched the realities of care. This was particularly powerful when families were either questioning or reluctant to use opioid medication. As one nurse described:

*“Sometimes when families leave, we will give it [morphine] and then tell them after the fact that we gave it because we still have orders for it. Sometimes they are [angry] and sometimes later they come around and realize they needed it, especially if their respiratory distress returns after the morphine stated to wear off, and if you can let them see the before and after. So remember how good mom looked when you came in and how comfortable she was? And how now she’s not. And if you let me give her that again, we could take her back there to be comfortable.”*[RN02: 803–821]

*Reducing the potential for future regret:* These behaviors, described more often by nurses, identified that families needed to feel reassured, particularly around decision-making about the resident’s care. Sometimes this meant that nurses acquiesced to what the family wanted, especially when it came to transferring the actively dying resident to hospital. Many nurses saw this as a necessary, albeit unfortunate step that had to be taken, in helping families come to terms with the fact that the resident was dying. While nurses tried to negotiate and find common ground when making decisions with families about the care of the resident, they realized that respecting family choices and providing reassurance minimized the potential for future regrets in the family:

*“Sometimes it’s a path of least regret. So if the family really wants blood work to make sure we haven’t missed something, and if this person is just dying and not something we can fix, a poke at the end of life is not fantastic but I don’t think often the resident that got poked would rather that family have some peace of mind than that person dying and wondering what else we could have done.”* [RN02: 416–421]

*Keeping family comfortable while sitting vigil:* HCAs more often than nurses described behaviors that kept the family comfortable while sitting vigil when the resident was close to death. If family began to stay at the bedside round the clock once the resident was actively dying, HCAs would ensure the family was kept comfortable though such actions as providing food and beverages, extra chairs, blankets and a bed for them to stay overnight at the bedside:

*“We get what they need, you know we make sure we offer coffee. Sometimes they stay overnight. To make sure you know, they get blanket to sleep on, make sure they’re okay. We give, we give, we support family. With things like that. 199% we always support them. We offer coffee, we offer lunch. Breakfast we do.”* [HCA03: 1201–1222]

In addition to these concrete behaviors, HCAs described more subtle activities to ensure comfort by monitoring family needs such as taking cues from the family as to when to be present and when to “give them space” [HCA03: 2283] to allow time alone with the resident. They also tried to protect families from the aggression that was sometimes displayed by residents close to death:

*“Well sometimes they’re upset but like, I remember once this patient let the daughter come and visit and she swore at her and I have to say you know what, it’s just her dementia and she will calm down and maybe you could come back later. She said yes it’s okay, but she was crying but you know, it’s hard for the family.”* [HCA20: 234–238]

### Engaging with others

Key behaviors emerged demonstrating how nurses and HCAs experts engaged with others to achieve their overarching goal of achieving comfort for the resident with dementia who was dying. Experts in this study had the willingness and humility to recognize their need for assistance from other team members, and would identify appropriate resources in order to ensure that residents and their family members received optimal care:

*“And we’re great teams….Once we decide then it’s kind of a team approach, you know. I’ll look at the palliative care kit. They’ll call the family. You know what I mean. And so it’s just done with an ease that you know, there’s no drama.... the other nurse might take over the technical stuff. Like getting the sub cut line in and, you know getting all that ready to go. Where the other nurse who has an emotional connection to the family deals with that part. You know like deals with the family piece. I mean we’re not, you know, super nurses that we can do it all. There’s other talents that other people bring… And I think, that not so great palliative nurse, she didn’t involve anybody else. Like it was all her or nothing.”* [LPN08: 1066–1083]

As part of this care ethos, teamwork was highly valued. Nurses described the important value they placed on HCA assessment, judgment, and skills. They relied on and trusted HCAs to provide them with critical information regarding the resident. HCAs in turn reported that they felt valued and supported by their nurse colleagues to bring forward their observations and concerns:

*“But 100% rely on your health care aides. And they sometimes don’t now the signs. They know something’s changed. Yeah it’s a big part of in nursing homes. They’re our first defense.”* [LPN06: 245–247]

### Responding after the death has occurred

Experts described a series of behaviors that occurred after the death of a resident. These behaviors were not specific to the care of those residents who had died with dementia, but rather exemplified an ethos of care that permeated care provided to any resident who died. Nurses and HCAs described the importance of demonstrating, especially to the family, how important the resident had been to the staff and how they would be missed. This often meant that even before the resident had died, nurses and HCAs would come to the room to share stories with the family about the resident. This type of reminiscing and storytelling often extended throughout the vigil and continued after the death of the resident:

*“Actually I had one resident not too long ago who was passing away and her family was so grateful for everything and they even liked the fact that I went in there and I was talking to her. Even though she couldn’t respond to me… the nurses told me the last thing to go for residents is their hearing. So I’m told, still talking to them, and reminding them of things that happened. Family appreciate that and then they tell me stories about [the resident]. Say really nice things like that before they go”* [HCA04: 97–104].

Both nurses and HCAs spoke of attending the funerals of residents who had died as a means of showing the family support and to acknowledge their own grief and loss. The death of a resident was seen as a hard process because of the length of time many residents were in their care. Celebrating the resident’s life acknowledged the close attachments that developed between staff and residents in long-term care:

*“I know for a fact it’s not my family but then you look after this, like my Mom, like a lady, or any, a man you know, your Dad. It’s like, like part of your family. Then when [it is] the end of their life you know it’s really sad, you cry. Like the last person that died, I cried so bad. Cried so bad. I even slept right beside, not really slept like lie down right beside her, hug and everything. I said don’t go, don’t’ go…”*[HCA03: 90–108]

Ensuring emotional support for the family was a critical element of the care experts provided leading up to and immediately after the death of a resident.

*“A lot of the support staff spend a lot of time with our residents too. They’ll go in with the families and do the same family bonding and supporting them in their way and also after the resident has passed away. I find the support staff are awesome. They’ll go in and ask the families what they need and help them pack up and do all those kinds of things and always give them that reassurance that we’re here no matter what, you know.”* [RN014: 685–692]

There were also a set of behaviors that honored the resident after death through facility rituals and through the actions of HCAs as they cared for the body. Expert nurses and HCAs recognized the solemnity of the death and tempered their behaviors to respect this. The described speaking in hushed tones when preparing the body for removal from the facility. While the experts themselves may not have instituted the practices, they certainly spoke with pride about them:

*“So we also have this thing here when somebody passes…the quilt …it’s to put over the coffin and we walk them out the front door because that’s how they came in. So you have a health care aide that walks the resident. That was adopted about a year ago I guess. So they came in the front they’re going to go out the front.”*[HCA07: 975–978]

Experts in this study supported their colleagues and other residents in the NH after the resident with dementia had died. Many spoke of the ripple effect a resident’s death can have in a facility highlighting the impact on other residents. HCAs described how at times their work can be both challenging and sad “because we are like a family or friend to the resident” [HCA03: 95]. As a result, there was a clear need to support each other.

### Having a positive attitude toward Care of the Dying

Nearly all of the nurses and HCAs described a defined role for themselves in the care of the dying. This role differed slightly based on scope of practice, yet both groups articulated a clear vision of what constitutes a good death, and their moral obligation to achieve it:

*“My role as a nurse is to make sure this woman dies in comfort. And I’ve identified that she’s dying and this is what I’m going to do about it whether you like it or not. And I think there needs to be more of it. There needs to be more people like me who will say, I’m not afraid.”* [RN05: 1088–1091]

*“I just think that, you know, no one’s on the earth forever and I just think everybody’s time does come to an end. So I’m here to make that as comfortable as possible.”*[HCA11: 84–86]

Advocacy played a large part of this defined role; for nurses this often meant advocating on behalf of the resident to either the physician or family; for the HCA it meant speaking to the nurse or the family regarding their observations of the resident.

*“Be involved. Be the caregiver. Be the advocate. Be the family. Like just be involved. Don’t just be on the outside looking in and asking your health care aides on the unit, oh did they void today? Be active in the dying process with the resident. As a nurse on the unit, be aware of all your residents and be aware of change. Say to heck with the paperwork. Human people are more important than that piece of paper. Just do your charting, just be active in the dying process with them along with the family.”*[LPN06: 877–890]

Part of this defined role was also to serve as a mentor to other members of the team as a means of strengthening capacity in the care of dying residents. Experts recognized that some of their colleagues were not comfortable with caring for dying residents and used these opportunities as teachable moments. When they were called to step in and provide care, nurses and HCAs both led by example:

*“Being in the job for so many years, sometime the new ones that come on, doesn’t really get what it’s like to really spend that time with a person who is dying. Because I think within themselves, they’re scared. Because I see nurses say, if somebody is dying, they’ll say well I cannot handle the situation. I will, send somebody else to help. But with me, being here so long, I see everything. I don’t know everything but I see. So I go through it with them and say, this is what’s going to happen. This is what’s going to take place…we’ll work together. I’ll work with you.”*[HCA01: 1051–1058]

A particular aspect of this role was described as bearing witness to the resident’s death. Being with the resident and staying at their bedside when no one else was present, was a critical aspect to the definition of a good death for both nurses and HCAs. This often involved making time or finding time to provide this one-to-one care. In some instances, HCAs even came in on their days off to ensure the resident had someone with them when they were actively dying:

*“My role is to be there for that person and to do whatever we can to keep that person as comfortable as possible. I feel that nobody should die alone. My way of looking at it is when we come into the world, we are welcomed in. And that when we’re going, somebody should be there to help us along the way.”* [LPN013: 396–399]

## Discussion

The critical behaviors described by nurses and HCAs in this study provides emerging evidence of best practices in care of those with dementia and their families, particularly near the end of life. All of these behaviors served to achieve the overarching goal of resident comfort—a salient outcome articulated in the European Association for Palliative Care white paper defining optimal palliative care in older people with dementia [19] and the World Health Organization’s global perspective on palliative care [20].

Participants in this study were able to gather and analyze information about the resident, identify those individuals with care needs, and implement or alter their actions to address those needs. These behaviors are consistent with Tanner’s [21] definition of clinical judgement, defined as, “an interpretation or conclusions about a patient’s needs, concerns, or health problems, and/or the decision to take action (or not), use or modify standard approaches, or improvise new ones as deemed appropriate by the patient’s response” (p. 204). In exercising their clinical judgement, participants drew upon two types of knowledge: general knowledge informed by experience and theory, and particular knowledge of the individual resident for whom they were caring [22]. This is a unique characteristic of the NH environment where staff can form significant bonds with residents, owing to longer length of stay of many residents and allows them to be more in tune with deviations from resident normal behaviors [23].

Participants in this study stressed the importance of caring for people with dementia in ways that privileged their uniqueness as individuals and emphasized their preferences. This stance is consistent with Kitwood’s [24] person-centered care approach which posits that individuals are much more than their diagnosis of dementia. Expert care providers acknowledged the importance of maintaining a sense of who the person was before they had dementia right through to the end of their life. While part of this involved ensuring care practices resonated with the needs and preferences of the resident, it went beyond that to recognizing the importance of maintaining a human connection through the resident’s death and in supporting the family in the immediacy after death. This requires comfort not only with dying and the non-verbal nature of many people living with dementia, but also values the profound impact of presence. In shifting from “doing to, to being with” [25], nurses and HCAs are a caring presence, and create an atmosphere of shared humanness and connection [26] that extends beyond the need for verbal exchange.

Expert care providers also saw the resident and family as the unit of care; a model consistent with the palliative approach yet one that often is still not embraced within the long-term care culture [27]. Nurses and HCAs in this study respected the expertise of family regarding the resident and valued their knowledge and contribution to the resident’s plan of care. Research has shown that families typically feel excluded from care and are not seen as allies or even in need of care themselves [28]. Exclusion of family members’ perspectives is problematic, given that they often act as proxies for residents, providing important information about changes in behavior or condition [29]. McPherson and colleagues (2008) assert that as health status deteriorates, reliance on family caregiver assessments should assume greater, not lesser importance.

Affirmation of the important roles that others play in the care of residents was also manifest through the value study participants placed on team work, and their acknowledgement of HCA knowledge and expertise. Nurse experts in this study recognized the skill and knowledge HCAs possessed, and understood the importance of communicating with them to access their knowledge about the health and well-being of residents. That nurse experts exemplified behaviours that value the unique roles HCA play in the NH environment is significant. First, communication has been highlighted as a core component of providing person-centred care [30], and research demonstrates that ineffective communication between nurses and HCAs, including nurses failure to use HCA knowledge result in negative resident outcomes [31, 32]. Second, feeling needed and valued have been identified as factors contributing to health care aide satisfaction with their work in long-term care settings caring for people with dementia (Sung, Change, & Tsai, 2005). Our findings thus resonate with that of Madden and colleagues [33] who identified that the ability to provide efficient resident care is positively influenced through nurse-HCA interactions that are grounded in cooperative communication, respect, and collegiality. Experts acknowledged that it could be difficult at times to engage families, and that skill was required to find common ground with them. Experts also recognized, however that it was equally difficult for families to witness the changes in the resident brought on by the progression of dementia and to reconcile the fact the person was dying. Death was something new and unfamiliar to many family members, and a clear goal of experts in this study was to support families in this journey. An important part of working with families was normalizing for them the terminal nature of dementia. Given studies have found that families often have limited understanding of dementia is a disease that one dies from [34], and that nursing home residents with advanced dementia may thus be likely to receive burdensome interventions at the end of life [35, 36], work that nurses and HCAs engage in to help families understand the clinical trajectory of dementia is warranted. One of the surprising findings in our study was that part of this support was often to acquiesce to the family as a means to minimize future regret regarding decisions made about the resident’s care. Regret has been examined in the context of end-of-life decision-making [37] and is seen as a process whereby a negotiation between affective (i.e., emotional) and analytical thoughts need to be weighted. In instances of end of life decisions, the need to minimize emotional distress and the view that one is making “the wrong decision” is often achieved through the sense that all options had been exhausted before declining further treatments [38]. At times, this may be likened to what Bauer and colleagues [27] termed “keeping the family happy”.

Participants in this study demonstrated a positive attitude toward the dying—a disposition identified in other studies examining the nature of expert nursing practice in end of life care [10, 12, 15]. There is consensus in the literature that death related fears negatively impact health care providers’ attitudes about providing care to those nearing the end of life [39, 40]. Our findings further support that the attitudes held by those attending to the dying influences the quality of care they are able to provide [39].

Study participants recognized the impact that the death of a resident had not only on family, but on staff and other residents in the long-term care facility as well. The need to provide emotional support to colleagues and other residents is consistent with what Wowchuk (2004) described as ‘care of the institutional family’. The relationships that care providers have with residents can be emotionally intense, and the emotional attachments and multiple losses that that those in long term care experience necessitates the need for administrators to develop strategies to support care providers in their expression of grief [41].

### Limitations

While this study adopted several strategies to ensure a rigorous approach to the methods employed, there are limitations that must be acknowledged. The qualitative nature of the study precludes the generalizability of the findings. The majority of participants were female and attitudes towards death and dying may be influenced by gender [42]. While there was some ethnic variability in our sample, a predominantly Caucasian viewpoint is represented. Further studies with greater gender and ethnic diversity may help to further understand critical behaviors in the care of residents dying with dementia.

## Conclusion

Providing optimal care to those dying with dementia in the NH setting requires nurses and HCAs to not only become comfortable with a palliative approach to care but also to recognize the subtle shifts in the progression of the disease. By striving to maintain a resident’s sense of self and through the adoption of philosophies of care that value presence, openness, being proactive and inclusion of families in care, expert nurses and HCAs create an environment which allows the possibility of a comfortable death for the resident. Understanding these care behaviors will assist clinicians, educators, and employers in delineating practices to guide future care of residents dying with dementia.

## Data Availability

The dataset generated and analyzed during the current study is not publicly available since consent to make the data publicly available was not sought from participants during the consenting process.

## Abbreviations

HCA: Health care aide
NH: Nursing home
